# Efficacy of injectable versus topical formulation of ivermectin against *Anopheles stephensi* mosquitoes feeding on different body locations of treated Holstein calves

**DOI:** 10.1186/s13071-025-07225-9

**Published:** 2026-01-31

**Authors:** Staci M. Dreyer, Todd Molden, Marc L. Bauer, Colleen Pfaff, David J. Smith, Jefferson A. Vaughan

**Affiliations:** 1 Department of Biology, University of North Dakota, Grand Forks, North Dakota USA; 2 Science Department, Valley City State University, Valley City, North Dakota USA; 3 Department of Animal Sciences, North Dakota State University, Fargo, North Dakota USA; 4 Food Animal Metabolism Research Unit, Agricultural Research Service, U.S. Department of Agriculture, Fargo, North Dakota USA

**Keywords:** *Anopheles stephensi*, Ivermectin, Formulation, Residual malaria

## Abstract

**Background:**

Malaria is a major public health concern and is transmitted to humans by the bite of infected *Anopheles* mosquitoes. One strategy to reduce populations of zoophagic *Anopheles* (i.e., likely to feed on other animals as well as humans) is the use of systemic veterinary parasiticides. The most widely systemic parasiticide used for this purpose is ivermectin. Ivermectin is available for livestock in two formulations; injectable and topical “pour-on.” The purpose of this study was to evaluate the survival and fecundity of a zoophagic species, *Anopheles stephensi*, when fed on calves treated with different ivermectin formulations.

**Methods:**

Three groups of four dairy calves were used; calves in one group received a single subcutaneous injection of commercial ivermectin, calves in another group were treated topically once with pour-on ivermectin, and the third group was left untreated. At various times after treatment, groups of mosquitoes were fed simultaneously on different parts of the body to determine if feeding location of mosquitoes influenced the efficacy of treatment. Engorged mosquitoes were maintained for 7 days to monitor survival and fecundity.

**Results:**

Both formulations significantly reduced *An. stephensi* survival and fecundity for up to 9 and 14 days, respectively, following treatment of calves. Topical formulation of ivermectin applied to the back of the calves significantly reduced the survival of *An. stephensi* that fed on the back for up to 23 days after treatment, but not for mosquitoes that fed concurrently on the belly or the leg of the same calves, suggesting that a portion of topically applied ivermectin may remain at the site of application. Mosquitoes were less likely to feed on topically treated calves, implying that topical application may confer some mild repellency.

**Conclusions:**

Determining the body location(s) where zoophagic *Anopheles* mosquitoes feed on livestock (e.g., legs) will allow targeted application and methods (e.g., foot baths) for more efficient use of topical formulations of ivermectin as part of an integrated zoophagic vector management strategy.

**Graphical Abstract:**

**Figure:**
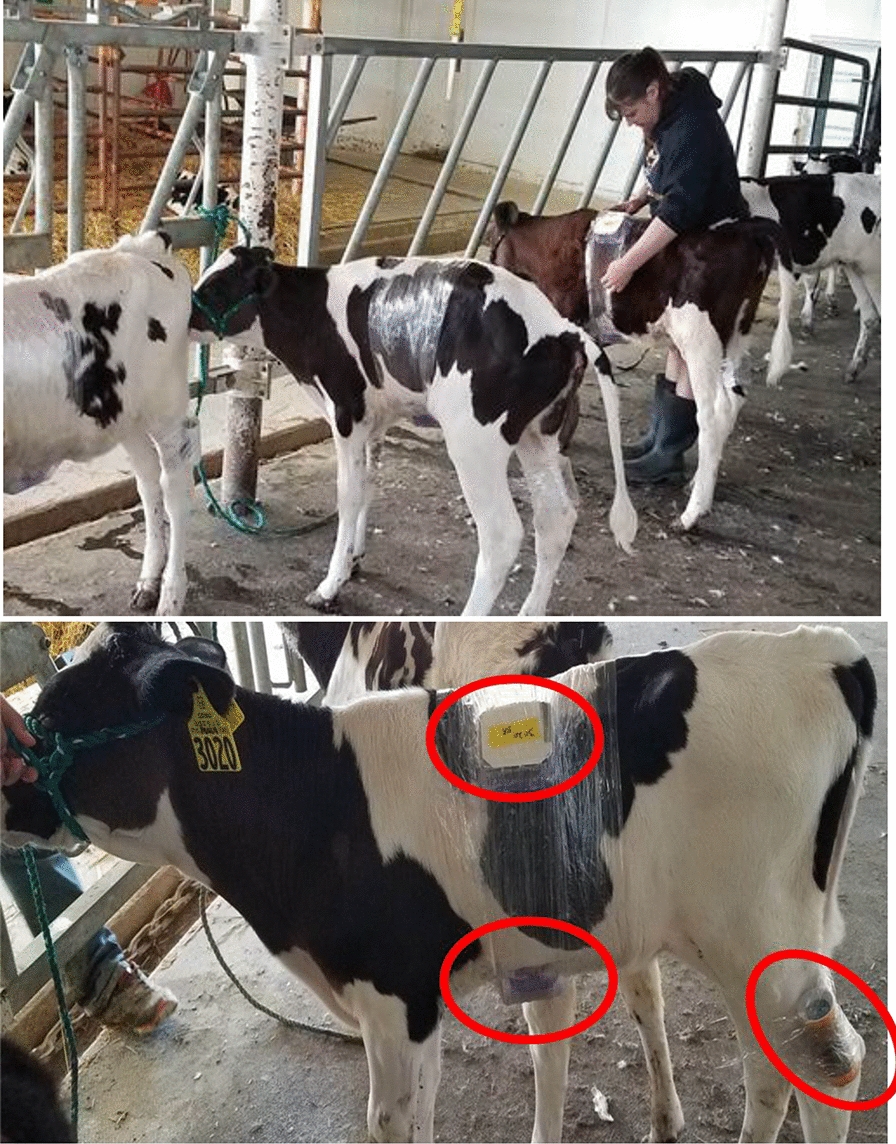

**Supplementary Information:**

The online version contains supplementary material available at 10.1186/s13071-025-07225-9.

## Background

Malaria is one of the most severe public health concerns worldwide, with almost 263 million cases and 597,000 deaths reported in 2023 [1]. To combat the disease and reach eventual eradication, multiple vector control strategies will be needed. One strategy to reduce populations of zoophagic *Anopheles* (i.e., likely to feed on other animals as well as humans) is the use of systemic veterinary parasiticides. By treating livestock with certain drugs, mosquitoes that feed on treated livestock can be killed and/or sterilized. In this way, livestock treatment could reduce vector populations and help address the problem of residual malaria [2–5]. The most widely used systemic parasiticide tested in livestock for this purpose is ivermectin [6–18].

Formulation of drugs and insecticides play an important role in their effectiveness and utility. Ivermectin is widely available for use in livestock in two formulations; subcutaneous injection and topical “pour-on” formulations. The purpose of this study was to evaluate survival and ovarian development in *Anopheles stephensi* after feeding at different anatomical locations on cattle treated with either subcutaneous injection or topical pour-on formulations of ivermectin. *An. stephensi* is zoophagic in its feeding preference [19] and the strain used (STE-2) is susceptible to ivermectin [20], making it an appropriate species/strain for study. Specific goals were to (1) compare how long residual insecticidal efficacy would last for each of the two formulations, (2) determine if the body location where mosquitoes fed influenced the residual efficacy of a treatment, (3) determine if treatments inhibited blood meal digestion and ovarian development of surviving mosquitoes, and (4) determine if treatments altered mosquito feeding behavior (i.e., repellency).

## Methods

### Mosquitoes and mosquito feeding containers

The STE2 strain of *An. stephensi* was obtained as eggs in 2003 from the U.S. Centers of Disease Control and Prevention through the MR-4 Program (BEI Resources, Manassas, VA USA) and has been in continuous culture for over 20 years in the University of North Dakota insectary with > 200 generations. There was a single introduction of the NYU strain (kind gift of Dr. Jerome Vanderberg) in 2005. Mosquitoes were reared at a photoperiod of 12-h light:12-h dark and a temperature of 26–28 °C. Eggs were hatched in trays of aged tap water. Larvae were maintained on fish food (Pond Sticks, Tetra U.S.^®^ Blacksburg, VA USA) ground to a fine powder in an electric coffee grinder. Pupae were separated en mass from late instar larvae using an ice water technique. Briefly, larvae and pupae were sieved and backwashed into a separatory funnel with ice cold water. Immobilized, the insects quickly separated by their relative buoyancy (i.e., larvae sink, pupae float). Larvae were placed back into rearing trays to resume development. Pupae were placed into cages to complete metamorphosis. Emergent adults were provided with water and a sugar source.

Mosquito containers for feeding on the back and belly of calves were modified from rectangular 330 mL polypropylene food storage containers with locking lids (Supplementary Fig. S1). Portions of the lid and sides were cut out and screen mesh affixed into place with a glue gun to; (1) allow mosquitoes access to the skin of the calves and (2) provide ventilation to minimize condensation buildup on the plastic during feeding. The bottom of each container was lined with clean filter paper, replenished after each feeding. A separate hole at one end of the container was covered with two layers of elastic dental dam with perpendicular slits cut to allow the introduction of mosquitoes into the chamber via aspirator. This design worked well for feeding mosquitoes on the relatively flat surfaces of back and belly, but not on a curved surface such as the leg. Mosquito containers for feeding on the legs of calves were modified from cylindrical cardboard shipping tubes (ca. 14-cm long with 5.5 cm inside diameter), capped at each end and similarly modified with screen and dental dam. At 2 h before feedings were to take place, female mosquitoes, 3–7 days posteclosion, were aspirated from holding cages into the feeding containers (20–70 per container). Feeding containers containing mosquitoes were placed in a large plastic storage container with locking lid and transported by automobile to the North Dakota State University Animal Sciences Dairy Cattle Research and Teaching Center located in Fargo, North Dakota (~1-h travel time) where cattle treatment and mosquito feedings were conducted.

### Cattle treatment, mosquito feeding, and postfeeding monitoring

A total of 12 Holstein heifer calves (6-week-old, 45–80 kg) were used, each with its own uniquely numbered ear tag. A preliminary mosquito feeding was conducted on each calf to establish baseline information on mosquito feeding rates, postfeeding survival, and to optimize the logistics and procedures for mosquito transport, feeding, and calf handling. The following week, each calf was randomly assigned to one of three groups (four calves per group). One group received a subcutaneous injection of 1% ivermectin (Durvet^®^, 1 mL per 50 kg body weight [= 0.2 mg ivermectin per kg BW]) under the loose skin of the neck region. Another group received ivermectin pour-on, applied along the dorsal midline from neck to the base of the tail (Durvet^®^, 1 mL per 10 kg body weight [= 0.5 mg ivermectin per kg BW]). Doses were as per recommendation on the product label. A control group of calves was left untreated. Throughout the study, each calf was housed individually in its own hutch (PolyDome^®^ Calf Nursery, Litchfield, MN USA) to prevent them from rubbing against or licking one another which can be a potential source of cross-contamination between treatments [21, 22].

Mosquito feedings were conducted in the mornings (9:00–11:00 h, local time) on days 2, 5, 9, 14, and 23 after treatment. Calves were led into the barn and hitched to a post using rope halters. Prior to initiating mosquito feedings, three small areas (*ca.* 10 × 10 cm) on each calf were shaved using a rechargeable livestock clipper—a patch near the dorsal midline, a patch on the belly, and a patch above the hind hock (tibial joint). On each calf, mosquitoes were fed simultaneously on the three shaved areas. Feeding containers for the back and belly were secured into place by encircling the torso several times with plastic food wrap and similarly for the hind leg (Supplementary Fig. S1b–d). Mosquitoes were allowed to feed for 15–20 min, after which the plastic wrap was cut with scissors, and the containers carefully removed from the calves. Feeding containers were oriented in the larger transport container in such a way that engorged mosquitoes rested on a nearly vertical surface. This placement allowed the copious amount of postfeeding diuretic fluid produced by engorged *Anopheles* to be directed away from the resting area and minimized the loss of mosquitoes becoming trapped within excreta during transport. Mosquitoes were transported back to the University of North Dakota insectary where they were released from feeding containers by carefully opening each container within the confines of a larger screen-mesh rearing cage. Fully engorged mosquitoes were transferred into 0.5 l cardboard recovery cages; unfed and partially fed mosquitoes were discarded. Engorged mosquitoes were maintained thereafter at a photoperiod of 12-h light:12-h dark, at a temperature of 26 °C, and had continuous access to cotton soaked in a 10% sucrose solution. Cages remained stationary and were not rotated during this time. Mosquito mortality was assessed every 24 h by counting and removing the dead mosquitoes from each recovery cage. At the end of 7 days, surviving mosquitoes were counted and dissected to evaluate blood meal digestion and ovarian development. Blood meal digestion was scored either positive (i.e blood present in midgut; blood meal not digested) or negative (i.e no blood visible in midgut; blood meal fully digested). Ovarian development was scored as fully gravid (ovarioles fully developed), half gravid (ovaries enlarged but ovarioles not fully developed), or not gravid (small ovaries with no ovariole development) [23].

### Plasma concentration of ivermectin

Approximately 15 ml of blood were taken from the jugular vein of each of the treated calves at days 2, 5, 9, 14, and 23 following treatment. Blood was collected in Vacutainer^®^ blood collection tubes coated with EDTA, centrifuged, and the plasma was removed and frozen in a −80 °C freezer until analysis at the U.S. Department of Agriculture, Agricultural Research Service facility in Fargo, North Dakota. Briefly, internal standard (4 ng D2-Ivermectin; Toronto Research Chemicals, Toronto, CA) was added to extraction tubes, the solvent (20 µL) allowed to evaporate under N_2_, and plasma (0.2 mL) added. Thereafter, 200 µL of room-temperature acetonitrile were added with a 5 s vortex. Samples were allowed to incubate at 40 °C for 1 h and were then centrifuged at 3273 × *g* for 5 min. Aliquots (200 µL) of each extract were transferred to glass autosampler vials and 10 µL volumes were analyzed by liquid chromatography–mass spectrometry (LC–MS/MS; Waters TQS) in the positive mode using ion transitions 893.8 $\to$ 145.4 and 893.8 $\to$ 113.3 for ivermectin and 894.7 $\to$ 309.4 for D2 ivermectin determination (transitions from ammonium adducts). Samples were injected onto a Waters Acquity BEH C_18_ column (2.1 × 50 mm; 1.7 µm) and eluted with an isocratic mobile phase of 5 mM ammonium formate with 0.05% formic acid (25%) and acetonitrile with 0.05% formic acid (75%). All samples were extracted and quantified in triplicate. Calibration curves were constructed from blank plasma fortified with 1, 2, 5, 10, 20, 50, and 100 ng/mL of native ivermectin and 20 ng/mL of D2 ivermectin and extracted in an identical manner to samples. The limit of quantitation for the plasma ivermectin was 1 ng/mL.

### Data analysis

Mosquito survivorship across treatment and body location were examined using Kaplan–Meier survival analyses and the log rank test. Plasma ivermectin concentrations in calves treated with injectable versus topical formulations were compared with repeated measures analysis of variance (ANOVA). Probit regression was used to estimate dose-mortality relationships (e.g., LC_50_ values). To examine if ivermectin treatments affected mosquito feeding behavior, the proportion of mosquito engorgement that occurred on treated and untreated calves was compared by computing odds ratios. Differences in rates of blood meal digestion, and ovarian development were analyzed among treatments and body positions using chi-square analysis. Data sorting and analyses were conducted using Microsoft Excel (Microsoft Corp., Redmond, WA, USA), IBM SPSS Statistics (Armonk, NY, USA), and Statistix (Analytical Software, Tallahassee, FL, USA). A 0.05 level of significance was used throughout.

## Results

A total of 12 calves were exposed to a total of 9629 *An. stephensi* mosquitoes—1407 mosquitoes during a pretreatment period (averaging 117 mosquitoes per calf per exposure) and 8222 mosquitoes on days 2 (*n* = 1926), 5 (*n* = 1673), 9 (*n* = 1098), 14 (*n* = 2146), and 23 (*n* = 1379) post-treatment, averaging 134 mosquitoes per calf per exposure. Mortality data (but not engorgement data) for mosquito feedings conducted on day 2 post-treatment was excluded from analysis because unacceptably high mortality (56%, *n* = 464) occurred during the transport of engorged mosquitoes fed on the control calves. This mortality was owing to suboptimal handling techniques of the feeding chambers during the first few hours after blood feeding. Slight adjustments in handling technique (as described above) solved the problem. For days 5, 9, 14 and 23 post-treatment, overall mean (± SD) percent survival of engorged mosquitoes within the feeding chambers during transport from barn to insectary was 98.9 ± 4.1% (*n* = 4595). Separate cohorts of mosquitoes were used at each exposure. On day 9, we were not able to rear enough mosquitoes to conduct mosquito feedings concurrently on all three body locations of the 12 calves and therefore, feedings were conducted only on the belly and leg of the calves. The overall percentage of mosquitoes that engorged on days 5, 9, 14 and 23 post-treatment was 71% (*n* = 6296), averaging 97 engorged mosquitoes per calf per exposure.

### Duration of mosquitocidal activity

**Fig. 1:**
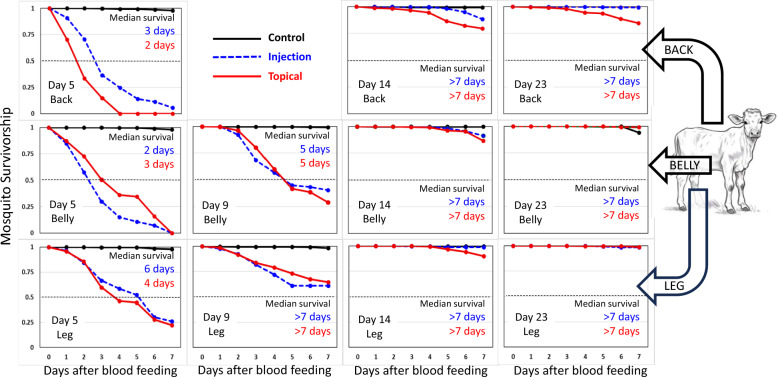
Survival curves for cohorts of *Anopheles stephensi* mosquitoes fed simultaneously on the back, belly, and leg of calves treated with injectable formulation of ivermectin (*n* = 4), topical formulation of ivermectin (*n* = 4), and untreated control calves (*n* = 4)

After each feeding, mosquito survival was monitored for 7 days (Fig. 1). The overall 7-day survival of mosquitoes fed on untreated calves (all time points and feeding locations combined) was 97.7 ± 3.2% (*n* = 1692) and the median survival exceeded the 7-day holding period. The average 7-day post-feeding survival of mosquitoes fed on the backs and bellies of ivermectin-treated calves at 5 and 9 days after treatment was 4.0 ± 6.5% (*n* = 777) and median survival ranged from 2 to 5 days (Fig. 1, Table 1). However, the average 7-day postfeeding survival of mosquitoes fed on the hind legs of the same treated calves at days 5 and 9 (25.6 ± 31.4%; *n* = 431) was significantly higher (cx^2^ = 42.4, *P* < 0.001) and median survival ranged from 4 to over 7 days (Fig. 1, Table 1). By day 14 after treatment, the mosquitocidal activity of ivermectin-treated calves had dissipated markedly and median survival of mosquitoes fed on treated calves extended beyond the 7-day holding period (Fig. 1, Table 1). Even so, survival analysis indicated that mosquitoes fed on the back of calves treated with topical ivermectin formulation at 23 days after treatment had significantly lower 7-day survival (69.0 ± 20.9%, *n* = 98) than did mosquitoes fed on either the bellies (98.2 ± 2.1%, *n* = 107) or hind legs (96.8 ± 2.4%, *n* = 99) of the same calves at the same time (cx^2^ = 59.3, *P* < 0.001, log rank statistic = 7.1; Table 1). The duration of mosquitocidal activity resulting from ivermectin treatment of calves was greatest with the topical formulation when mosquitoes fed on the same anatomical location where the topical formulation had been applied (i.e., the back).

**Table 1:** Statistical comparisons (log rank test) among Kaplan–Meier survival curves for *Anopheles stephensi* fed on calves receiving two different formulations of ivermectin (rows) and on three different body locations (columns). Median survival expressed as days (*n* = number of fed mosquitoes)

Day after treatment	Treatment	Body location		
		Back	Belly	Leg
5	Injection	3 days (*n* = 155)	2 days (*n* = 157)	6 days (*n* = 193)
	Topical	2 days (*n* = 98)	3 days (*n* = 132)	4 days (*n* = 82)
	Log rank test	*P* < 0.001 *L* = 6.82	*P* < 0.001 *L* = −4.11	*P* = 0.261 *L* = 1.12
9	Injection	^a^	5 days (*n* = 125)	> 7 days (*n* = 74)
	Topical	^a^	5 days (*n* = 110)	> 7 days (*n* = 78)
	Log rank test		*P* = 0.301 *L* = −1.03	*P* = 0.231 *L* = 1.43
14	Injection	> 7 days (*n* = 129)	> 7 days (*n* = 197)	> 7 days (*n* = 76)
	Topical	> 7 days (*n* = 159)	> 7 days (*n* = 161)	> 7 days (*n* = 154)
	Log rank test	*P* < 0.001 *L* = 5.86	*P* = 0.056 *L* = 1.91	*P* = 0.003 *L* = 2.98
23	Injection	> 7 days (*n* = 146)	> 7 days (*n* = 167)	> 7 days (*n* = 58)
	Topical	> 7 days (*n* = 98)	> 7 days (*n* = 107)	> 7 days (*n* = 99)
	Log rank test	*P* < 0.001 *L* = 7.10	*P* = 0.378 *L* = −0.86	*P* = 0.586 *L* = −0.54

^a^ Insufficient numbers of *An. stephensi* mosquitoes were available to conduct mosquito feedings on all three body locations

### Plasma concentration of ivermectin and mosquito mortality

**Fig. 2:**
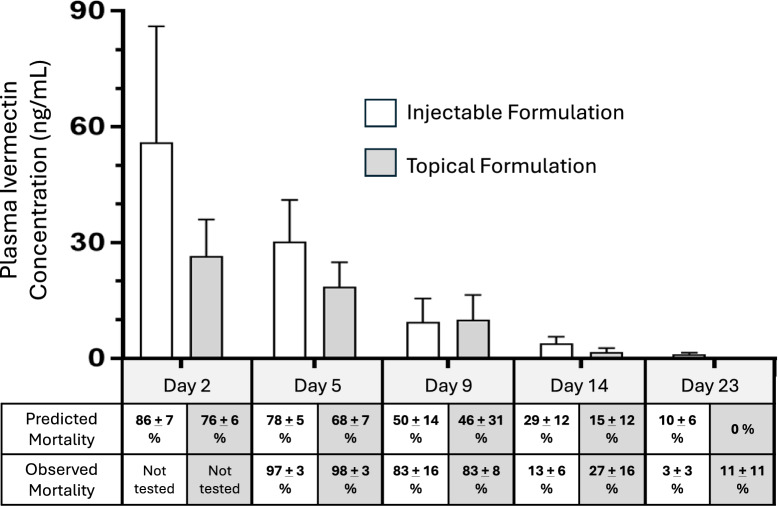
Average (± SD) concentration of ivermectin (ng/mL) in the plasma of 6- to 8-week-old Holstein calves at 5, 9, 14, and 23 days after subcutaneous injection (white bars) or topical application of ivermectin (shaded bars). Predicted mortality of *Anopheles stephensi* mosquitoes as a function of plasma ivermectin is based on the dose–response equation of Dreyer et al. [20]. Actual mortality was quantified in mosquitoes fed directly on the calves from which plasma samples were collected

**Fig. 3:**
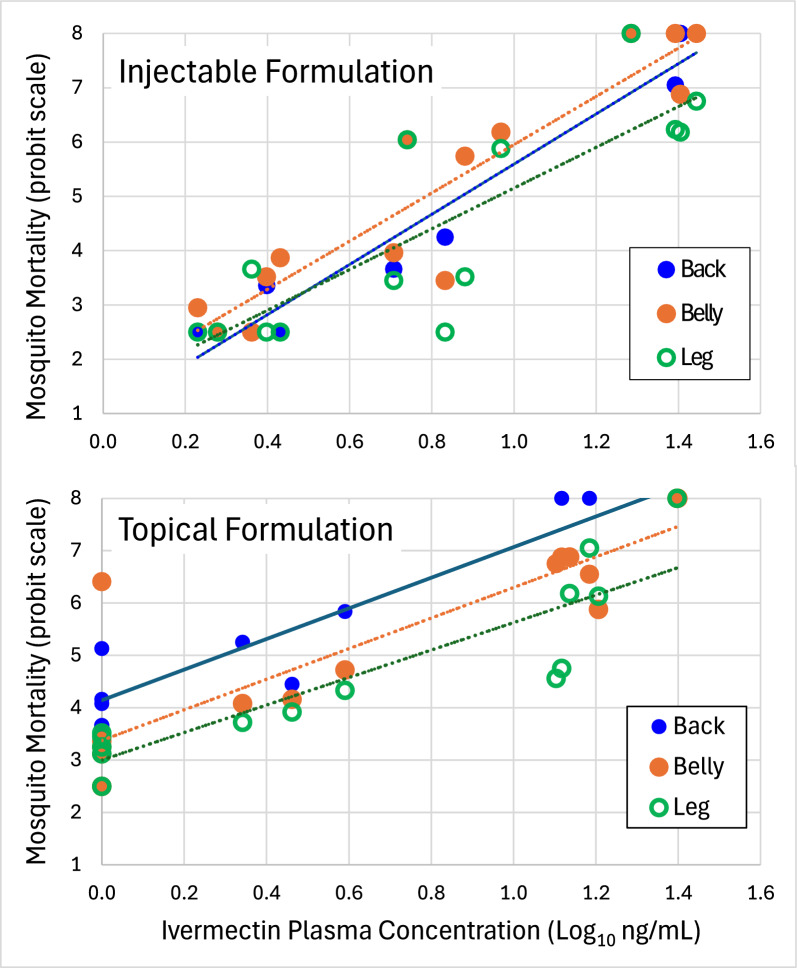
Relationship between plasma concentration of ivermectin (log_10_ ng/mL) at the time of mosquito feeding and subsequent mosquito mortality (probit scale) 7 days after feeding

At days 2 and 5 after treatment, the average concentrations of ivermectin in the plasma were higher in calves administered subcutaneous injections than in calves administered topical formulation (Fig. 2, *F* = 7.46, df 1, 30, *P* = 0.04). However, the decline in plasma concentration of ivermectin between days 2 and 9 after treatment occurred slower in calves treated with topical formulation (elimination rate constant = 0.137, half-life = 5.1 days) than in calves treated with injectable formulation (elimination rate constant = 0.246, half-life = 2.8 days). Plasma concentrations of ivermectin became negligible by 23 days after treatment. Predicted percentage mortalities at each time point were calculated as a function of plasma ivermectin concentration on the basis of the dose–response equation of Dreyer et al. [20] which utilized membrane feeding of technical grade ivermectin and the same strain of *An. stephensi* as used in these trials. For many time points, the observed mortalities were greater than predicted mortalities based on the dose–response equation (cx^2^ tests, *P* < 0.05). For example, 9 days after treatment, the average plasma concentrations in injected-treated and topically treated calves were both near the theoretical LC_50_ concentration (*ca*. 7 ng/ml). Yet, mosquitoes feeding on treated calves experienced > 80% mortality. For topically-treated calves (Fig. 2, grey bars), observed mortalities exceeded predicted mortalities for each of the four time points examined. For injected calves (Fig. 2, white bars), this happened only at days 5 and 9 post-treatment. To examine this more closely, six dose-mortality curves were constructed corresponding with each formulation and body location of mosquito feeding. The three dose–mortality curves for mosquitoes fed on injected calves (i.e., fed on back, belly, leg) clustered together whereas the three curves for mosquitoes fed on topically-treated calves were more widely spread (Fig. 3). Each dose-mortality curve was subjected to probit analysis to estimate corresponding LC_50_ values (Table 2). The overall oral toxicity of calves that received injectable formulation of ivermectin (LC_50_ = 7.3 ng/ml) was statistically the same as that of technical grade ivermectin administered via membrane feeders (LC_50_ = 7.0) (Table 2). The overall oral toxicity of calves that received topical formulation (LC_50_ = 3.7) was significantly greater than that of both injectable and technical grade ivermectin (i.e., nonoverlapping confidence intervals). This difference was owing to the exceptionally low LC_50_ value observed for mosquitoes that fed on the back of topically-treated calves (LC_50_ = 2.0 ng/ml, Table 2).

**Table 2:** Oral toxicity of ivermectin to *Anopheles stephensi* mosquitoes, expressed as LC_50_ values (ng/ml) with 95% confidence limits in parentheses, comparing mosquitoes fed on calves treated with different commercial formulations of ivermectin versus mosquitoes fed technical grade ivermectin in membrane feeders [20]

Method of feeding	Ivermectin formulation	Fed on back	Fed on belly	Fed on leg	Body locations combined
Live host	Injectable	7.8 (5.5, 12.4)	6.5 (5.0, 9.2)	8.1 (5.4, 11.7)	7.3 (6.2, 8.7)
	Topical	2.0 (1.2, 3.6)	3.7 (1.8, 8.3)	6.1 (4.4, 8.5)	3.7 (2.6, 5.1)
Membrane feeder	Technical grade	–	–	–	7.0 (5.2, 8.6)^a^

^a^Data from Dreyer et al. [20]

### Sublethal effects

The numbers of surviving mosquitoes fed on treated calves at day 5 after treatment were too low to allow meaningful examination of blood meal digestion and ovarian development, and information on sublethal effects were only feasible with mosquitoes fed at days 9, 14, and 23 after treatment (Table 3). A total of 2572 mosquitoes were dissected and examined for blood residue in the midgut and ovarian development. At days 9 and 14 after treatment, a lower proportion of mosquitoes fed on injection-treated calves (64% and 66%, respectively) and topically treated calves (30% and 75%, respectively) had digested their blood meals compared with mosquitoes fed on untreated calves (> 99%, χ^2^ > 166, *P* < 0.0001). At day 23 after treatment, there were no differences in the proportion of blood meals digested (> 95%, *P* > 0.07) between mosquitoes fed on treated versus untreated calves. Similarly, at days 9 and 14 after treatment of calves, a lower proportion of mosquitoes that fed on injection-treated calves (36% and 79%, respectively) and topically treated calves (26% and 75%, respectively) had fully developed ovaries compared to mosquitoes fed on untreated calves (> 90%) (χ^2^ > 136, *P* < 0.0001). On day 23 after treatment, there was no difference between mosquitoes fed on topically treated versus untreated calves (*P* = 0.236). However, there was a significantly lower proportion of mosquitoes with fully developed ovaries that fed on injection-treated calves (54%) than on untreated calves (62%) (χ^2^ = 5.2, *P* < 0.02). Notably, ovarian development in control fed mosquitoes at day 23 (62%) was much lower than in control fed mosquitoes at days 9 (90%) and 14 (93%). The reasons for this are speculative but may have been due to an immune response of calves to the intense mosquito feedings that occurred during the previous weeks. Lowered ovarian development in mosquitoes fed on control calves at day 28 may have influenced the statistical comparison between treated and control.

**Table 3:** Bloodmeal digestion and ovarian development in surviving *Anopheles stephensi* fed on treated and untreated calves

Day after treatment	Treatment	Number mosquitoes dissected	Proportion with digested bloodmeals (95% CI)	Statistical difference from control	Proportion with fully developed ovaries (95% CI)	Statistical differences from control
9	Injection	31	0.64 (0.38, 0.91)	*P* < 0.001 (*χ*^2^ = 106.5)	0.36 (0.04, 0.69)	*P* < 0.001 (*χ*^2^ = 87.6)
	Topical	36	0.30 (0.07, 0.52)	*P* < 0.001 (*χ*^2^ = 151.1)	0.26 (0, 0.53)	*P* < 0.001 (*χ*^2^ = 71.1)
	Control	284	0.99 (0.98, 1.00)	–	0.90 (0.83, 0.97)	–
14	Injection	338	0.66 (0.51, 0.82)	*P* < 0.001 (*χ*^2^ = 210.2)	0.79 (0.71, 0.88)	*P* < 0.001 (*χ*^2^ = 25.1)
	Topical	348	0.75 (0.63, 0.87)	*P* < 0.001 (*χ*^2^ = 176.8)	0.75 (0.62, 0.89)	*P* < 0.001 (*χ*^2^ = 38.7)
	Control	566	1.00 (0.99, 1.00)	–	0.93 (0.90, 0.96)	–
23	Injection	264	0.95 (0.91, 1.00)	*P* = 0.073 (*χ*^2^ = 3.2)	0.54 (0.40, 0.67)	*P* = 0.023 (*χ*^2^ = 5.2)
	Topical	361	0.99 (0.98, 1.00)	*P* = 0.280 (*χ*^2^ = 1.2)	0.62 (0.50, 0.73)	*P* = 0.236 (*χ*^2^ = 1.4)
	Control	344	0.97 (0.93, 1.00)	–	0.62 (0.56, 0.69)	–

### Blood feeding and repellency

**Fig. 4:**
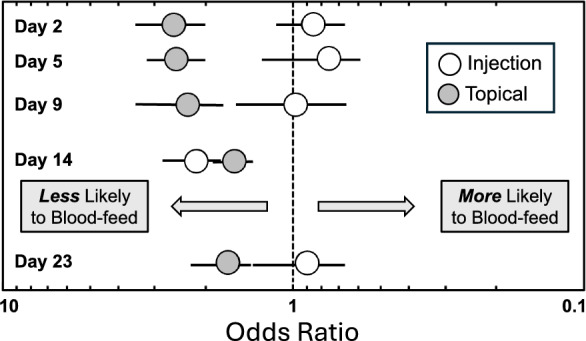
Odds ratios (with 95% confidence intervals) comparing the proportion of mosquitoes that blood-fed over a 23-day period on untreated calves (represented by the central dotted line; odds ratio = 1) versus calves treated with subcutaneous injection (open circles) or topical formulation (shaded circles) of ivermectin. Odds ratios greater than one indicate mosquito repellency

Overall blood feeding success during the post-treatment period was 71.1% (*n* = 6296), but success varied according to treatment (Fig. 4). Throughout the 23-day post-treatment period, mosquitoes were less likely to feed on calves treated with topical ivermectin (61.6%, *n* = 2623) than on untreated cattle (76.5%, *n* = 2848) (*χ*^2^ = 137.7, *P* < 0.0001; odds ratio = 2.03, 95% confidence limits 1.81, 2.28). There was no overall difference between mosquito feeding rates on ivermectin-injected calves (74.5%, *n* = 2680) versus untreated calves (76.5%, *n* = 2848) (*χ*^2^ = 3.0, *P* = 0.083; odds ratio = 1.12, 95% confidence limits 0.99–1.26). As the experiment progressed, the odds of mosquitoes feeding on topically treated calves improved but remained significantly less than the odds of mosquitoes feeding on untreated calves (Fig. 4). Mosquitoes feeding on calves treated with injectable ivermectin showed no difference in feeding rate than mosquitoes feeding on control calves, except on day 14 post-treatment (Fig. 4); however, the feeding rate returned to normal (odds ratio = 1) by day 23 post-treatment (Fig. 4).

## Discussion

This study compared the duration of mosquitocidal activity in calves after treatment with two widely available livestock formulations of ivermectin—injectable and topical. Doses were chosen on the basis of the recommended dosages listed on the product labels and engorged mosquitoes were monitored daily for a week after they fed on treated and untreated calves. Both formulations of ivermectin reduced the 7-day survival of *An. stephensi* mosquitoes feeding on treated calves for at least 9 days after calf treatment (Fig. 1, Table 1) which is consistent with other cattle-based studies examining the residual efficacy of subcutaneously-injected ivermectin against *Anopheles* mosquitoes [6–18]. By day 14 after calf treatment, the mosquitocidal efficacy of calves receiving subcutaneous injections was negligible (Fig. 1). However, calves receiving topical formulation retained significant mosquitocidal activity on days 14 and 23, but only against mosquitoes that fed near the vicinity where the drug had been applied—i.e., on the back. Mosquitoes feeding on the other locations (belly and hind legs) were not affected. The localized effect observed in this study may reflect differences between formulations in how ivermectin is absorbed and dispersed into the bloodstream. Both injectable and topical formulations displayed rapid absorption and plasma concentrations peaked at 2 days following treatment. Interestingly, the initial plasma levels of ivermectin were higher in calves receiving subcutaneous injections at a lower dose (0.2 mg/kg BW) than in calves receiving topical application of a higher dose (0.5 mg/kg BW) (Fig. 2). This is similar to earlier reports. Pharmacokinetics studies in cattle comparing drug absorption of different formulations of ivermectin and related drugs, moxidectin and doramectin have shown that topically formulations typically result in lower bioavailability (i.e., lower plasma concentration) compared with injection or oral routes of administration. The lowered bioavailablity of topically-applied ivermectin may reflect differences in the metabolism and rates of ivermectin excretion between topically-treated versus injected cattle. For example, Herd et al. [24] reported that cattle treated with a topical formulation of ivermectin (0.5 mg/kg BW) produced significantly higher concentrations of ivermectin in their feces in the first few days following treatment than the cattle treated with a lower dose of injectable ivermectin (0.2 mg/kg BW). Even so, plasma levels of ivermectin for topically-treated cattle in this study and in others [25–29], have been shown to persist longer than for injected cattle. Because ivermectin is highly lipophilic, it was hypothesized that topically applied ivermectin may become sequestered in the skin and released into the blood more slowly. Indeed, Sallovitz et al. [30] demonstrated that the related drug, moxidectin, when topically applied, is sequestered at high concentrations within the epidermis and dermis near the site of application and in areas of high adipose concentration. Presumably, if ivermectin behaves in a similar manner, then dermal sequestration could account for the longer-lasting mosquitocidal efficacy exhibited by topically treated calves (Fig. 1). For example, even as drug concentrations in the general circulation dropped below efficacious levels, mosquitoes may have still encountered dermal deposits of ivermectin during probing and the drug may be absorbed through the thin cuticle of the mosquito fascicle. Alternatively, cutaneous ivermectin may have become metabolized, producing mosquitocidal metabolites [31] that were not detected by our mass spectrometry techniques. The notion of mosquitocidal metabolites sequestered in the dermis of topically-treated calves is supported by our observation that the acute oral toxicity of topically-treated calves (LC_50_ = 3.7 ng/mL, Table 2) was greater than that for injected calves and for experimental membrane feedings using pure ivermectin (LC_50_ = 7.0 to 7.3 ng/mL). Regardless of the mechanism(s) involved, the mosquitocidal efficacy of both formulations exceeded that predicted from our in vitro laboratory assays (Figs. 2 & 3, Table 2).

A significant proportion of mosquitoes that survived feeding on ivermectin-treated calves failed to digest their blood meals, failed to complete vitellogenesis and, as a result, failed to develop gravid ovaries. These sublethal effects were observed on days 9 and 14 after treatment but had largely diminished by day 23 after treatment (Table 3). Previous studies with cattle have also shown that for a period of time after the mosquitocidal effects of treatment have worn off, ivermectin-treated animals can still produce sublethal effects that disrupt the reproductive biology of anopheline mosquitoes. For example, Lyimo et al. [9] reported that blood digestion (as measured by the amount of hematin defecated by *An. arabiensis* after blood feeding) was significantly less in mosquitoes when fed on ivermectin-injected cattle versus normal cattle. The reduced blood meal digestion occurred in ivermectin-fed mosquitoes for up to 9 days after cattle had received treatment, but day 12 full blood digestion in engorged mosquitoes had returned to normal. Similarly, Lyimo et al. [9] and Fritz et al. [6] reported significantly lower egg production in *An. arabiensis* and *An. gambiae s.l.* fed on cattle treated with subcutaneously injected ivermectin. Dreyer et al. [11] observed reduced blood meal digestion and ovarian development in field-collected *An. albimanus* that survived feeding on an ivermectin-treated heifer for up to 7 days after treatment. By day 14, sublethal effects on *An. albimanus* had dissipated. The sublethal effects of ivermectin in reducing *Anopheles* reproduction can supplement the drug’s mosquitocidal effect and further contribute to reducing overall vector populations.

Although the topical formulation provided longer-lasting efficacy than the injectable formulation, topical formulations may have some disadvantages compared with injectable formulation. For example, it was noted that mosquitoes were less likely to feed on topically treated calves than on untreated calves (Fig. 4). This “repellent effect” was not observed for ivermectin-injected calves. Repellency is not a desirable feature for systemic insecticides and to our knowledge, this is the first report of potential repellency of ivermectin against mosquitoes. This feature may be unique to the topical pour-on formulation of ivermectin. A recent study with Zebu cattle subcutaneously injected with ivermectin reported no repellency against *An. arabiensis* feeding [17]. Similarly, no repellency was noted in *An. albimanus* mosquitoes feeding on a heifer treated with subcutaneously injected ivermectin [11]. Topical ivermectin repellency was not observed for host-seeking *Culicoides* midges when sticky traps were mounted onto the backs of cattle treated with topical ivermectin [32]. Although a significant repellent effect was noted for the topical formulation in this study, the proportion of mosquitoes feeding on the backs of topically treated calves was still quite high (ca. 60–70%) and the repellency was outweighed by the significantly longer residual efficacy of the formulation (Fig. 1, Table 1).

In terms of ecotoxicity, both formulations result in ivermectin residues being excreted in the feces which can harm nontarget arthropod decomposers (e.g., dung beetles) [33]. Topical formulations have been shown to produce a surge of excreted ivermectin in the feces [24], which could lead to a higher, albeit transitory, level of ecotoxicity in topically treated cattle versus injected cattle. In addition, the topical formulation has a longer withdrawal time than the injectable formulation. According to the package insert, cattle treated with topical ivermectin must not be treated within 48 days of slaughter for human consumption, whereas cattle treated with injectable ivermectin must not be treated within 35 days of slaughter. However, in practical terms, when considering the use of ivermectin in livestock for *Anopheles* control, withdrawal times are probably irrelevant since ivermectin has been proven safe for human consumption and is presently part of mass drug administration trials in people for malaria control [34, 35].

Even though there may be some disadvantages of topical over injectable formulation, an important consideration of the basic strategy—i.e treating livestock for malaria control—is the acceptance of people to adopt the strategy. In the case of ivermectin, it is likely more practical and cheaper for owners to treat their livestock with a “pour-on” formulation than it is to inject the cattle. Topical formulations do not require syringes. Thus, a topical formulation may be preferable simply due to its ease of use. The other advantage is the longer duration of efficacy when mosquitoes were fed at the site where the topical formulation was applied (i.e., the back, see Fig. 1). Duration of efficacy could be maximized if the material was applied to the site where the target mosquito prefers to feed. For example, *An. gambiae*, *An. arabiensis*, and *An. funestus* are reported to feed habitually on the feet and ankles [36]. *Aedes aegypti*, *An. atroparvus*, and *An. albimanus* often feed around the head, neck, and shoulders [36–38]. Although these studies are based on mosquito feeding on humans, it is reasonable to assume that feeding preferences also exist with zoophagic mosquitoes on livestock. Thus, if it is determined that a target vector species feeds preferentially on the back or ears of cattle, then topical treatment should be along the back or head. Conversely, if the target vector species preferentially feeds on the belly or legs of livestock, then spraying the animal further down on the body or setting up foot baths for livestock to walk through could optimize the treatment. Coupled with its ease of application and duration of efficacy, topical formulations of ivermectin should be considered for use as part of an integrated zoophagic vector management strategy.

## Conclusions

Both injectable and topical formulations of ivermectin were effective at killing mosquitoes and reducing ovarian development. Despite producing a mild repellent effect, the long-lasting residual effects of topically applied ivermectin, coupled with its ease of application, makes the topical “pour-on” formulation of ivermectin an attractive option for livestock-mediated vector control. Information on where mosquitoes preferentially feed on cattle can be used to optimize where on the body topical ivermectin would be most efficiently applied to livestock (e.g., foot baths). Such knowledge may also be used to reduce the amount of ivermectin needed per treatment.

## Supplementary Information

**Supplementary Material 1. **Table S1. Topical formulation of ivermectin. Comparison of serum concentrations (ng/mL) and corresponding mosquito (*Anopheles stephensi*) mortality after feeding on 6- to 8-week-old Holstein calves at various intervals after were treated with pour-on ivermectin (Durvet^®^) along the calves’ dorsal midline. At each interval for each calf, groups of mosquitoes were fed simultaneously on the back, belly, and hind leg near the hock.

**Supplementary Material 2. **Table S2. Injectable formulation of ivermectin. Comparison of serum concentrations (ng/mL) of ivermectin and corresponding mosquito (*Anopheles stephensi*) mortality after feeding on 6- to 8-week-old Holstein calves at various intervals after calves received a single subcutaneous injection of commercial ivermectin (IvoMec) in the neck region. At each interval for each calf, groups of mosquitoes were fed simultaneously on the back, belly, and hind leg near the hock.

**Supplementary Material 3.**Figure S1. Experimental feeding of *Anopheles stephensi* mosquitoes on calves. **A** Laboratory-reared mosquitoes were aspirated into sturdy, screen-top cassettes modified from food storage containers (@ 120 per cage) and transported by automobile to the dairy facility. **B** The back, belly and hind leg of calves were shaved and the mosquito cassettes were strapped into place using plastic food wrap. **C** Mosquito feedings were conducted on back, belly and leg for multiple animals stanchioned in place. **D** Mosquitoes were allowed to feed for 15 to 30 min, then the plastic wrap was cut with scissors, cassettes carefully removed, and transported back to the laboratory for processing.

## Data Availability

All data generated during this study are included in this published article and its supplementary information files.
