# Supplementary figures and images for: Efficacy of injectable versus topical formulation of ivermectin against Anopheles stephensi mosquitoes feeding on different body locations of treated Holstein calves
Source: Parasit Vectors. 2026 Jan 31;19:62. doi: 10.1186/s13071-025-07225-9 (PMC12865956; doi:10.1186/s13071-025-07225-9)

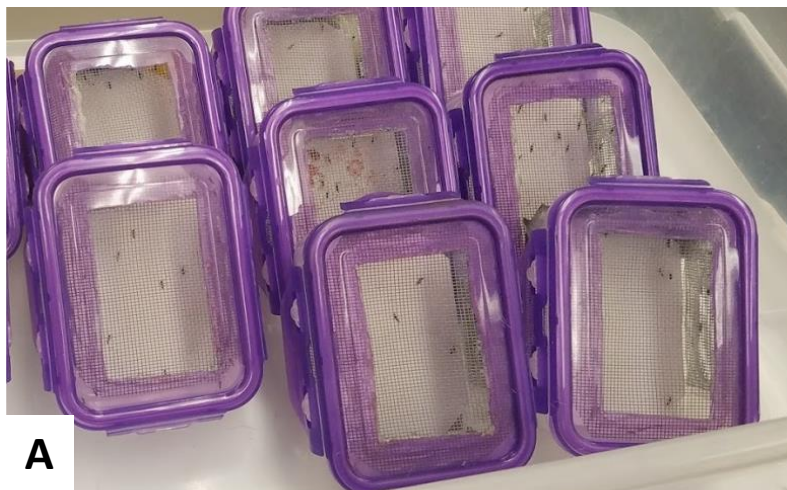

A

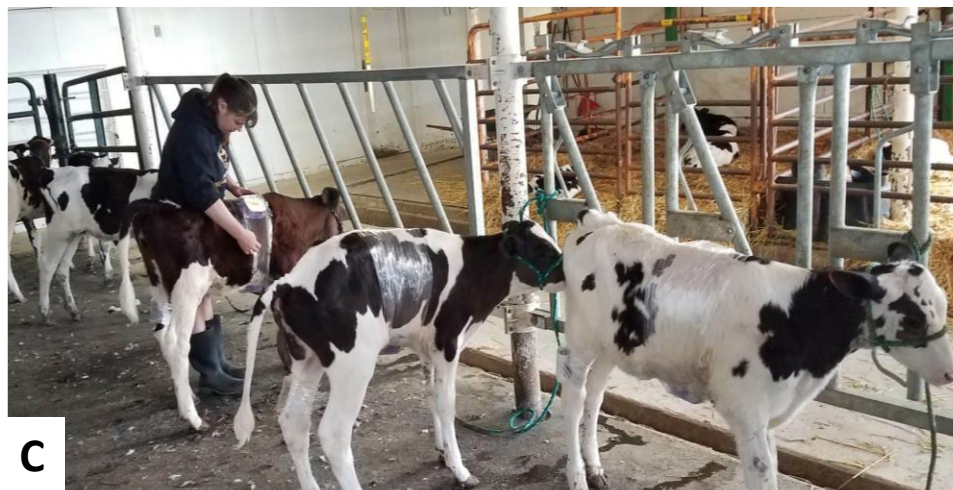

C

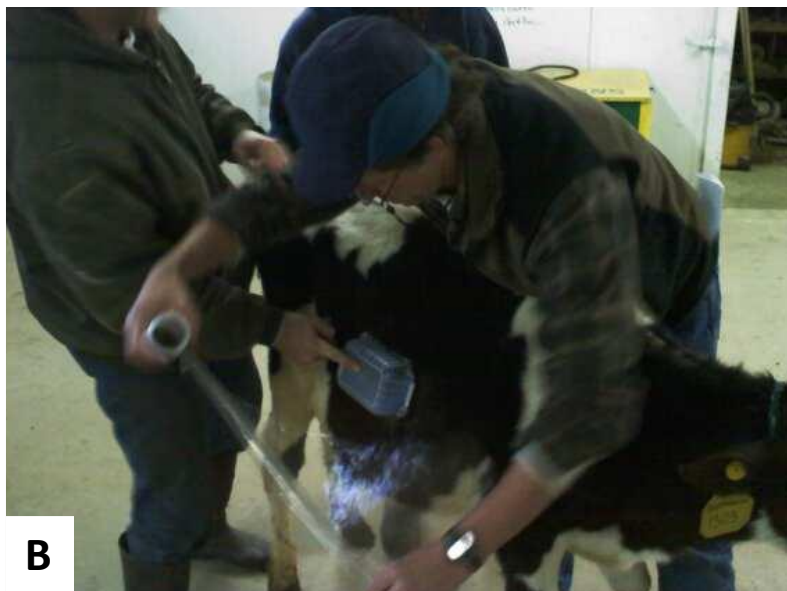

B

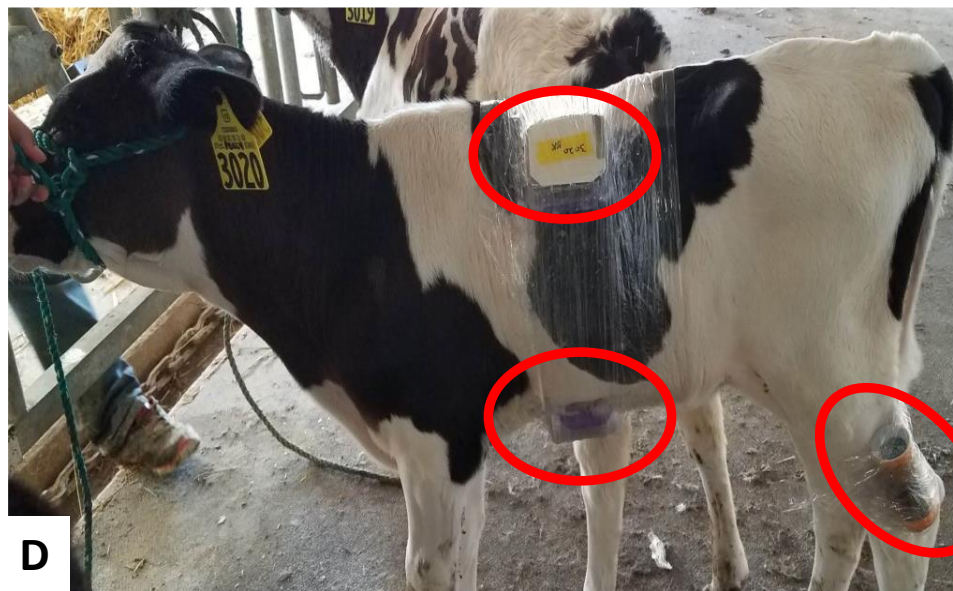

D

Supplement: Supplementary file 1 — Supplementary Material 1. Table S1. Topical formulation of ivermectin. Comparison of serum concentrations (ng/mL) and corresponding mosquito (Anopheles stephensi) mortality after feeding on 6- to 8-week-old Holstein calves at various intervals after were treated with pour-on ivermectin (Durvet®) along the calves’ dorsal midline. At each interval for each calf, groups of mosquitoes were fed simultaneously on the back, belly, and hind leg near the hock. [file 13071_2025_7225_MOESM1_ESM.pdf]
