# Supplementary material for: Efficacy of injectable versus topical formulation of ivermectin against Anopheles stephensi mosquitoes feeding on different body locations of treated Holstein calves
Source: Parasit Vectors. 2026 Jan 31;19:62. doi: 10.1186/s13071-025-07225-9 (PMC12865956; doi:10.1186/s13071-025-07225-9)
Supplement: Supplementary file 2 — Supplementary Material 2. Table S2. Injectable formulation of ivermectin. Comparison of serum concentrations (ng/mL) of ivermectin and corresponding mosquito (Anopheles stephensi) mortality after feeding on 6- to 8-week-old Holstein calves at various intervals after calves received a single subcutaneous injection of commercial ivermectin (IvoMec) in the neck region. At each interval for each calf, groups of mosquitoes were fed simultaneously on the back, belly, and hind leg near the hock. [file 13071_2025_7225_MOESM2_ESM.docx]

**Table S1.** Topical Formulation of ivermectin (Durvet®). Serum concentrations (ng/mL) of ivermectin in 6- to 8-week-old Holstein calves at various intervals after receiving pour-on ivermectin along the calves’ dorsal midline. and corresponding mosquito (*Anopheles stephensi*) mortality 7 days after feeding on Note: At each interval for each calf, groups of mosquitoes were fed simultaneously on the back, belly, and hind leg near the hock.

|  | Calf 3013 | | | | Calf 3014 | | | | Calf 3018 | | | | Calf 3019 | | | |
| --- | --- | --- | --- | --- | --- | --- | --- | --- | --- | --- | --- | --- | --- | --- | --- | --- |
| Day | IVM (ng/ml) | Back | Belly | Leg | IVM (ng/ml) | Back | Belly | Leg | IVM (ng/ml) | Back | Belly | Leg | IVM (ng/ml) | Back | Belly | Leg |
| 2 | 21.8 | 100% (n=7) | 83% (n=6) | 100% (n=4) | 21.5 | 100% (n=4) | 100% (n=55) | 100% (n=12) | 40.8 | 100% (n=12) | 100% (n=74) | 100% (n=18) | 21.9 | 96% (n=27) | 100% (n=36) | 100% (n=10) |
| 5 | 12.1 | 100% (n=23) | 97% (n=37) | 40% (n=5) | 24.1 | 100% (n=16) | 100% (n=31) | ^ | 24.0 | 100% (n=42) | 100% (n=31) | 100% (n=26) | 14.3 | 100% (n=17) | 94% (n=33) | 98% (n=51) |
| 9 | ND | NT* | 92% (n=25) | 20% (n=5) | 11.7 | NT | 96% (n=28) | 35% (n=17) | 12.7 | NT | 97% (n=31) | 88% (n=26) | 15.1 | NT | 81% (n=26) | 87% (n=30) |
| 14 | ND | 9% (n=34) | 5% (n=22) | 7% (n=28) | 1.2 | 60% (n=58) | 19% (n=47) | 11% (n=18) | 1.9 | 30% (n=37) | 21% (n=62) | 15% (n=40) | 2.9 | 80% (n=30) | 40% (n=30) | 26% (n=68) |
| 23 | ND | 18% (n=28) | 3% (n=35) | 0% (n=24) | ND | 20% (n=30) | 0% (n=25) | 3% (n=34) | ND | ^ | 0% (n=23) | 6% (n=18) | ND | 55% (n=40) | 4% (n=24) | 4% (n=23) |

*ND – Not Detectable

*NT – did not have enough *Anopheles stephensi*; used another more ‘ivermectin-tolerant’ mosquito species (*Aedes aegypti*) which only 4 of 74 (5%) were killed.

**^** No mosquitoes fed in this cage.
